# On an Easy Mode of Reducing a Dislocated Femur

**Published:** 1851-06

**Authors:** 


*On an easy mode of reducing a dislocated Femur.* By Dr. Mayr.—Dr. Fischer, of Cologne, published in *Casper's Wechenschrift*, No. 1, 1849, an account of his mode of reducing dislocation of the femur, and which consists in flexing the femur to an acute angle with the trunk, and impressing upon it gentle rotary movement while in a state of abduction, if dislocated on to the ileum. Dr. Mayr, without being then aware of this procedure of Dr. Fischer, resorted to it in a case that occurred lately to himself. A man aged thirty-one, dislocated his right femur upwards and backwards; and, after repeated attempts at reduction, even by the pulleys, had been made, the author was called in on the thirteenth day after the accident. After he had in vain tried the ordinary plan of extension and counter extension, he resorted to the following means: the opposite limb and the pelvis were fixed, the operator flexed the femur upon the trunk, and, passing one arm under the ham, while he grasped the calf with the other, he imparted rotary movements of gradually increased strength to the limb. As soon as he perceived a greater mobility of the head of the femur, he brought the limb into a state of strong abduction; and when, still continuing the rotation, the head had approached the æcetabulum, he was able, by a rapid and strong pull inwards, to slide it into its pan, which it entered with a loud noise. The gentle rotary movements mentioned by Fischer did not succeed here — all his force being required in their production, which may be probably due to the time the bone had remained unreduced.

The anatomical structure of the parts also recommends this procedure. In front of the thick edge of the æcetabulum the under surface of the ileum forms a perceptible depression; and if the directions given in the manuals are followed, of making the tractions obliquely from outwards inwards, and somewhat from behind forwards, be followed, the head of the bone must meet in this depression with a considerable obstacle to its progress. This sometimes even invincible obstacle appears to be avoidable by resorting to abduction.—*Casper's Wechenserift*, 1850, No. 9.—*Medical Times.*

